# RNF145 Promotes Hepatocellular Carcinoma Metastasis through Ubiquitination and Degradation of PCDH9

**DOI:** 10.32604/or.2025.073079

**Published:** 2026-01-19

**Authors:** Huan Zhang, Zhangwendi Xu, Yien Xu, Mao Li, Lingrui Liu, Caiyun He, Wenhuan Zhong, Jiliang Qiu

**Affiliations:** 1 State Key Laboratory of Oncology in South China, Guangdong Provincial Clinical Research Center for Cancer, Sun Yat-sen University Cancer Center, Guangzhou, 510060, China; 2 School of Medicine, Jishou University, Jishou, 416000, China

**Keywords:** Ring finger protein 145 (RNF145), protocadherin 9 (PCDH9), hepatocellular carcinoma, metastasis

## Abstract

**Objective:**

Ring finger protein 145 (RNF145), an E3 ubiquitin ligase, is significantly upregulated in hepatocellular carcinoma (HCC). However, its role in HCC remains unknown. The study aimed to investigate the functions and underlying mechanisms of RNF145 in HCC.

**Methods:**

The role of RNF145 in HCC was investigated using data from The Cancer Genome Atlas (TCGA) and *in vitro* experimental assays. Its oncogenic functions were assessed using the transwell migration assay and the wound-healing assay. The molecular mechanism was explored through protein immunoprecipitation and western blot analyses. Data from public databases were analyzed to correlate RNF145 expression with clinicopathological features. Univariate and multivariate Cox analyses established RNF145 as an independent prognostic factor. Subsequently, a prognostic nomogram was constructed.

**Results:**

RNF145 was upregulated in HCC. The expression level of RNF145 in HCC showed significant correlations with histological grade, pathological stage, and vascular invasion. Functionally, knockdown of RNF145 effectively abolished the migratory and invasive capacities of HCC cells. This pro-metastatic effect is mediated through the RNF145-driven ubiquitination and subsequent degradation of protocadherin 9 (PCDH9).

**Conclusion:**

Our findings confirm the significant upregulation of RNF145 in HCC and promote metastasis by facilitating PCDH9 ubiquitination and degradation, highlighting its role as a prognostic biomarker and a potential therapeutic target.

## 1 Introduction

Hepatocellular Carcinoma (HCC) is one of the most prevalent human malignancies and is the third most lethal tumor worldwide [1,2]. The high recurrence and metastasis rates of HCC contribute to its very low five-year survival rate [3,4]. A deeper understanding of the molecular mechanisms underlying HCC metastasis is urgently needed to facilitate the development of more effective treatment strategies.

Among the various protein degradation pathways, the ubiquitin-proteasome system is a highly specific, ATP-dependent intracellular degradation mechanism that plays a broad role in regulating key biological processes such as cell differentiation, proliferation, DNA damage response, and repair [5–7]. As a critical regulator of the ubiquitination process, E3 ubiquitin ligases play an indispensable role in regulating numerous cellular processes such as material synthesis, metabolism, the cell cycle, and metastasis [8–10].

Ring finger protein 145 (RNF145) is an E3 ubiquitin ligase mainly localized to the endoplasmic reticulum (ER) and plasma membrane [11,12]. It has been previously reported that RNF145 transcription is regulated by the sterol-responsive liver X receptor family, and that RNF145 is the main ubiquitin ligase promoting the degradation of 3-hydroxy-3-methylglutaryl-CoA reductase [11,13]. RNF145 is an important negative factor in the regulation of cholesterol synthesis and is associated with dyslipidemia [14,15]. However, the functions of RNF145 in cancer have not been reported in detail.

In this study, we aimed to determine the value of RNF145 in the diagnosis and prognosis prediction of HCC, and its relationships with clinicopathological features, immune cell infiltration and pathway. In this study, we therefore aimed to investigate the diagnostic and prognostic value of RNF145 in HCC, as well as its potential associations with clinicopathological features, immune cell infiltration, and relevant signaling pathways. Based on its known roles in regulated protein degradation, we hypothesized that RNF145 may influence HCC progression by modulating the ubiquitination of key metastasis-related proteins. This work seeks to clarify the function of RNF145 in HCC and explore its underlying molecular mechanisms.

## 2 Methods and Materials

### 2.1 Gene Expression Analysis of RNF145

The RNA sequencing data of HCC samples and normal tissues were downloaded from The Cancer Genome Atlas (TCGA) database (https://portal.gdc.cancer.gov/, accessed on 01 January 2025). The downloaded data were transformed from fragments per kilobase per million (FPKM) to transcripts per million (TPM) reads and then a log2 conversion was performed. The analysis of differential RNF145 mRNA expression between cancer and normal samples, and the visualization of the results, were carried out using the ‘ggplot2’ package (version 3.3.3) in R software (version 3.6.3). In addition, three datasets from the Gene Expression Omnibus (GEO) database (https://www.ncbi.nlm.nih.gov/geo/, accessed on 01 January 2025), namely GSE36376 [16], GSE62232 [17], and GSE76427 [18] were selected to perform similar analyses. Immunohistochemical staining images were used to evaluate the protein level of RNF145 and downloaded from the HUMAN PROTEIN ATLAS (https://www.proteinatlas.org/). It is open access to allow scientists both in academia and industry to freely access the data for exploration of the human proteome with the aim to map all the human proteins in cells, tissues, and organs using an integration of various omics technologies.

### 2.2 Diagnostic Value of RNF145 via Receiver Operating Characteristic (ROC) Analysis

To evaluate the diagnostic potential of RNF145 mRNA expression in HCC, we used ROC curves comparing tumor tissues with adjacent non-tumor or healthy liver samples. The area under the curve (AUC) was calculated to assess the sensitivity and specificity of RNF145 in discriminating HCC from non-malignant tissues. The optimal cutoff value was determined by maximizing Youden’s index (sensitivity + specificity − 1).

### 2.3 Clinicopathological Features

The clinical data of patients with HCC were downloaded from the TCGA database. Correlation analyses between RNF145 expression and histological grade, pathological stage, vascular invasion, albumin, alpha fetoprotein (AFP), age, sex, body mass index (BMI) and fibrosis ishak score were performed and visualized using the R software and ‘ggplot2’ package [19]. The criteria of Ishak score is as follows: 0, No fibrosis; 1, Fibrous expansion of some portal areas, with or without short fibrous septa; 2, Fibrous expansion of most portal areas, with or without short fibrous septa; 3, Fibrous expansion of most portal areas with occasional portal to portal (P-P) bridging; 4, Fibrous expansion of portal areas with marked bridging (P-P) and portal-central (P-C); 5, Marked bridging (P-P and/or P-C) with occasional nodules (incomplete cirrhosis); 6: Cirrhosis [20].

### 2.4 Survival Analysis

The prognostic value of RNF145 expression was assessed using the Kaplan–Meier plotter online database (http://kmplot.com/) and R software [21]. Patients were stratified into high-and low-expression groups to evaluate overall survival (OS), disease-specific survival (DSS), and progression-free survival (PFS). Survival differences were analyzed using Kaplan–Meier curves, with hazard ratios (HR), 95% confidence intervals (95% CIs), and log-rank *p* values calculated for each endpoint. Subsequently, the HRs with 95% CIs and *p* values for OS, PFS, and DSS across different subgroups were visualized in a forest plot generated with the ‘ggplot2’ package in R.

### 2.5 Univariate and Multivariate Cox Regression Analysis

To ascertain independent prognostic factors for HCC, we first conducted a univariate Cox regression analysis on two cohorts, which revealed a significant association between RNF145 expression and patient OS (*p* < 0.05). To verify the independence of this factor, a multivariate analysis was subsequently performed. RNF145 expression remained a statistically significant predictor of OS in this model (*p* < 0.05), confirming its status as an independent prognostic factor.

### 2.6 Genetic Alterations and DNA Methylation Analysis of RNF145 in HCC

The genomic profiles of RNF145 in HCC were analyzed using an online database, cBioPortal (https://www.cbioportal.org/) [22,23]. The DNMIVD database http://119.3.41.228/dnmivd/index/, accessed on 01 January 2025), a publicly available database, was used to analyze the DNA methylation status of RNF145 in HCC [24]. Moreover, the Pearson correlation analysis of the methylation level of the RNF145 promoter region and gene expression, as well as the scatter plot, were also completed through the online tools of the DNMIVD database.

### 2.7 Gene Ontology (GO) and Kyoto Encyclopedia of Genes and Genomes (KEGG) Analysis

To explore genes associated with RNF145, we performed a discrepancy analysis to find out the differentially expressed genes, using the ‘DESeq2’ package (Version 1.36.0) in R [25]. Then, a volcano plot and heat maps were used to visualize the data. GO and KEGG pathway analyses were then performed with the ‘clusterProfiler’ package (Version 4.4.4).

### 2.8 Gene Set Enrichment Analysis (GSEA)

Gene Set Enrichment Analysis (GSEA) is a computational method that determines whether a prior-defined gene set shows statistically significant differences between two biological states. All samples were divided into two phenotypes according to the median RNF145 mRNA expression level. GSEA was performed using the software GSEA_4.2.1, which was downloaded from the website (https://www.gsea-msigdb.org/gsea, accessed on 01 January 2025). For GSEA, we utilized two curated pathway databases: Reactome and WikiPathways. Reactome (https://reactome.org/) is a manually annotated database of biological pathways, providing detailed molecular interactions hierarchically organized pathways. WikiPathways (https://wikipathways.org/) is a community-driven platform offering diverse, up-to-date pathway models across multiple species. Both databases were applied to identify significantly enriched pathways (FDR < 0.05) in RNF145-associated HCC transcriptomes.

### 2.9 Downstream Genes Exploration Using a Venn Diagram

Based on previous research reports, RNF145 is a E3 ubiquitin ligase that mediates protein degradation. In our analysis, we identified 465 genes with negatively correlated expression (logFc < −1 and *p* < 0.05) using the data from the TCGA database. The mass spectrometry analysis indicating 401 potential proteins binding to RNF145 were obtained from the Supplementary materials of a previous study. A Venn diagram was generated with the ‘VennDiagram’ package (version 1.7.3) in R to determine the common targets between the two datasets.

### 2.10 Immune Infiltration Analysis via CIBERSORT

To evaluate the immune cell infiltration landscape in HCC stratified by RNF145 expression, we employed the CIBERSORT algorithm (version 1.06). RNA-seq data were normalized to transcripts per million (TPM) and uploaded to CIBERSORT (https://cibersortx.stanford.edu/) using the LM22 signature matrix, which defines gene expression profiles for 22 immune cell subtypes. Samples were dichotomized into RNF145-high and low groups based on median expression. By deconvoluting the immune composition via linear support vector regression (SVR), estimating the relative proportions of immune cell subsets in each sample. Results were filtered for significance (permutation *p* < 0.05). Differential infiltration between groups was assessed using Wilcoxon rank-sum tests, with false discovery rate (FDR) correction for multiple comparisons. Statistical analyses and visualization were performed using the ‘ggplot2’ package in R software.

### 2.11 Cell Lines, Culture Conditions and Tissue Samples

Hepatocellular carcinoma cell lines PLC and HCC-LM3 were authenticated and obtained from the State Key Laboratory of Oncology in South China (Sun Yat-sen University Cancer Center, Guangzhou, China). All cell lines were authenticated by short tandem repeat (STR) profiling and confirmed to be free of mycoplasma contamination prior to use in this study. These cells were cultured in Dulbecco’s Modified Eagle Medium (DMEM) (D8418, Sigma-Aldrich, St. Louis, MO, USA) with 10% fetal bovine serum (FBS) (FBS-UE500, Newzerum, New Zealand) at 37°C in a 5% CO_2_ environment. Eight pairs of cancer and adjacent tissues were acquired from patients with HCC who underwent resection at Sun Yat-sen University Cancer Center. All patients were pathologically confirmed as having primary HCC. The patients provided prior consent for the use of these clinical specimens in research and the Institutional Research Ethics Committee of the Sun Yat-sen University Cancer Center provided approval for the study (SZR2020-03).

### 2.12 Knockdown Using a Small Interfering RNA (siRNA)

Two siRNAs targeting RNF145 and a nonspecific scrambled siRNA sequence were purchased from the company (Kidan Biosciences Co., Ltd., Guangzhou, China). Cancer cells were transfected with siRNA using Lipofectamine 3000 reagent (L3000015, Invitrogen, Carlsbad, CA, USA). Complexes were formed in Opti-MEM, added to the cells, and after 4–6 h, the medium was replaced. Silencing efficiency was assessed by western blot after 48 h. The sequences of the siRNAs were as follows: siRNF145#1, 5^′^-AGGUGAUUAUUGAGUCUUGUATT-3^′^, siRNF145#2, 5^′^-UCCGUGCUGUAAGCCUUUGUUTT-3^′^.

### 2.13 Quantitative Reverse Transcription Polymerase Chain Reaction (qRT-PCR) Analysis

Total RNA was isolated from human hepatocellular carcinoma (HCC) cell lines (HCC-LM3 and PLC) using the EZ-press RNA Purification Kit (B0004D, EZBioscience, Roseville, MN, USA). Complementary DNA (cDNA) was synthesized from the extracted RNA using a 4× reverse transcription master mix (EZB-RT2GQ, EZBioscience, Roseville, MN, USA). Quantitative PCR was then carried out on a CFX96 real-time PCR detection system with the 2× SYBR Green q-PCR master mix (A0001-R2, EZBioscience, Roseville, MN, USA). Gene expression levels were normalized to GAPDH as an internal control and relative quantification was determined using the 2^−ΔΔCt^ method. The sequences of the primers used for qPCR were as follows:

RNF145 Forward: 5^′^-CTCCACAGGGGTTTGAGTGC-3^′^,

RNF145 Reverse: 5^′^-TTGAAGGGCTGATCCTGTCC-3^′^,

PCDH9 Forward: 5^′^-CCTGGAAGAACAGCAAGGTGA-3^′^,

PCDH9 Reverse: 5^′^-GGTGATGTTGGCATCCTGGT-3^′^,

Serine Dehydratase Like (SDSL) Forward: 5^′^-ATGGCTGCAGAGAAAGTCCT-3^′^,

SDSL Reverse: 5^′^-TCAGTTCTTGGCA GCTTCAG-3^′^,

Acireductone Dioxygenase 1 (ADI1) Forward: 5^′^-TGCCAGAGCTACATCAAGGC-3^′^,

ADI1 Reverse: 5^′^-ACCAGCAGCATCTTGTACCC-3^′^,

GAPDH Forward: 5^′^-CTCTGCTCCTCCTGTTCGAC-3^′^,

GAPDH Reverse: 5^′^-GCGCCCAATACGACCAAATC-3^′^.

### 2.14 Western Blot

Total cellular proteins were extracted from HCC-LM3 and PLC cells using Radioimmunoprecipitation assay (RIPA) lysis buffer (R0278, Sigma-Aldrich, St. Louis, MO, USA). 30 μg of total protein per sample was loaded onto 10% SDS-PAGE gels for electrophoretic separation. The separated proteins were then transferred onto PVDF membranes. The membranes were blocked with 5% non-fat milk in TBST for 1 h at room temperature, followed by incubation with the following primary antibodies overnight at 4°C: anti-RNF145 (1:1000, 24524-1-AP, Proteintech, Chicago, IL, USA), anti-PCDH9 (1:1000, 25090-1-AP, Proteintech, Chicago, IL, USA), anti-β-Actin (1:1000, 4970, CST, Danvers, MA, USA) and anti-Flag (1:1000, 14793, CST, Danvers, MA, USA). A chemiluminescence reagent kit (1705061, Bio-Rad, Hercules, CA, USA) was used to detect the protein bands. Protein band intensities were quantified using ImageJ software (Version 1.53t, National Institutes of Health, Bethesda, MD, USA).

### 2.15 Invasion and Migration Assays

For the transwell migration assay, 5 × 10^4^ HCC-LM3 and PLC cells were plated in the top chamber of each insert with a non-coated membrane. For the invasion assay, 10 × 10^4^ HCC-LM3 and PLC cells were plated in the top chamber of each Matrigel-coated insert. After incubation at 37°C for 18 h, cells that migrated or invaded were fixed and stained in dye solution containing 0.1% crystal violet and 20% methanol. The number of cells that had migrated or invaded was observed and counted using a BX-X700 microscope (Keyence, Osaka, Japan). For the wound-healing assay, 1 × 10^6^ HCC-LM3 and PLC cells were grown to confluence in 6-well plates. Wounds were scratched in each well using sterile micropipette tips, washed with 1× phosphate-buffered saline (PBS, pH 7.4) and cultured in DMEM without FBS to prevent cells from proliferating. The healing status was observed the wound from the same location every 8 h and the reduced width of the scratches represented the distance the cells had migrated. All cells above were used after transfection for 48 h.

### 2.16 Co-Immunoprecipitation (Co-IP)

The HDOCK is an online database (http://hdock.phys.hust.edu.cn/, accessed on 01 January 2025) for protein-protein and protein-DNA/RNA dockingbased on a hybrid algorithm of template-based modeling and *ab initio* free docking [26]. After the protein docking prediction was completed, co-immunoprecipitation was used to confirm the binding of the two. Flag-RNF145 was transfected into HCC-LM3 cells using the Lipofectamine 3000 reagent. Forty-eight hours post-transfection, the cells were lysed with lysis buffer. The lysates were then incubated overnight at 4°C with Anti-FLAG M2 magnetic beads (M8823, Merck, Darmstadt, Germany), respectively. Following incubation, the beads were washed six times with wash buffer. The eluted proteins were subsequently analyzed by Western blot.

### 2.17 Immunofluorescence Assay

HCC-LM3 Cells were seeded on coverslips. Following fixation and permeabilization, the cells were fixed with 4% paraformaldehyde (in 1× PBS, pH 7.4) for 60 min at room temperature and subsequently permeabilized with 0.1% Triton X-100 (in 1× PBS, pH 7.4) for 10 min. The samples were incubated overnight at 4°C with the primary antibody. The primary antibodies were as follows: anti-RNF145 (1:400, PA5-57718, Thermo Fisher Scientific, Waltham, MA, USA), anti-PCDH9 (1:500, 25090-1-AP, Proteintech, Chicago, IL, USA). Following three washes with 1× PBS pH 7.4, the cells were stained with the secondary fluorescent antibody for 1 h at 37°C. Nuclei were counterstained with 4^′^,6-Diamidino-2-Phenylindole (DAPI, D9542, Sigma-Aldrich, St. Louis, MO, USA). A Nikon CSU-W1 spinning disk confocal microscope (Nikon, Tokyo, Japan) was employed for image acquisition.

### 2.18 Cycloheximide (CHX) Chase Assay

To assess the half-life of PCDH9 protein, HCC-LM3 cells with RNF145 knockdown and control cells were treated with the protein synthesis inhibitor cycloheximide (239763-M, Sigma-Aldrich, St. Louis, MO, USA) at a final concentration of 100 μg/mL. Following CHX administration, total cellular proteins were harvested at designated time points (0, 5, 10, 15, and 20 h). The degradation kinetics of PCDH9 were then analyzed by Western blotting using an anti-PCDH9 antibody (1:500, 25090-1-AP, Proteintech, Chicago, IL, USA).

### 2.19 Statistical Analysis

R software (version 3.6.3) was used to conduct statistical analysis. Differences between groups were compared using the Wilcoxon rank-sum test or Student’s *t*-test, as appropriate. Correlations between RNF145 expression and clinical characteristics were calculated using the Spearman correlation test. Differences were considered statistically significant when *p* < 0.05.

## 3 Results

### 3.1 RNF145 Was Upregulated in HCC

**Figure 1:**
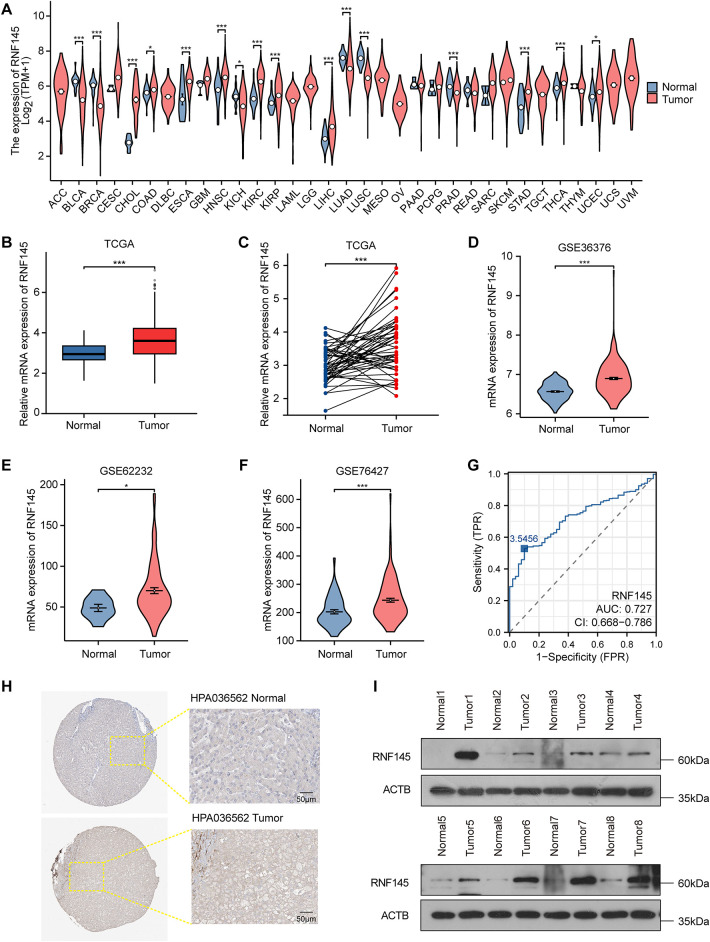
RNF145 was upregulated in HCC. The mRNA expression of RNF145 in 33 kinds of human cancers from the TCGA database (**A**). The mRNA expression level of RNF145 in HCC tissues compared with non-paired normal tissues (**B**) and paired normal tissues (**C**). The mRNA expression level of RNF145 in tumor and normal tissues from GSE36376 (**D**), GSE62232 (**E**) and GSE76427 (**F**). ROC curves of RNF145 for the diagnosis of HCC (**G**). Immunohistochemical staining images of RNF145 downloaded from the HUMAN PROTEIN ATLAS (**H**). Protein expression of RNF145 in 8 HCC tissues and paired normal tissues (**I**). *p < 0.05, ***p < 0.001

To investigate the role of RNF145, we first analyzed its mRNA expression across 33 human cancer types using TCGA database. Results revealed significantly elevated RNF145 expression in 10 cancer types compared to normal controls (Fig. 1A). Specifically, RNF145 mRNA levels were markedly higher than in both non-paired and paired normal tissues in HCC based on TCGA data (Fig. 1B,C). Consistent findings were obtained from the GEO datasets GSE36376, GSE62232, and GSE76427 (Fig. 1D–F). Receiver operating characteristic (ROC) analysis indicated high diagnostic value of RNF145 expression for HCC (AUC = 0.727) (Fig. 1G). Immunohistochemical images from the Human Protein Atlas further supported these results, showing stronger staining intensity and a higher proportion of RNF145-positive cells in HCC tissues than in normal samples (Fig. 1H). To further validate these findings, we performed Western blot analysis on proteins extracted from tumor and paired normal tissues of eight HCC patients, which confirmed the upregulation of RNF145 at the protein level (Fig. 1I). Collectively, these results demonstrate that RNF145 is consistently upregulated in HCC.

### 3.2 RNF145 Expression Correlated with Clinicopathological Features in HCC

**Figure 2:**
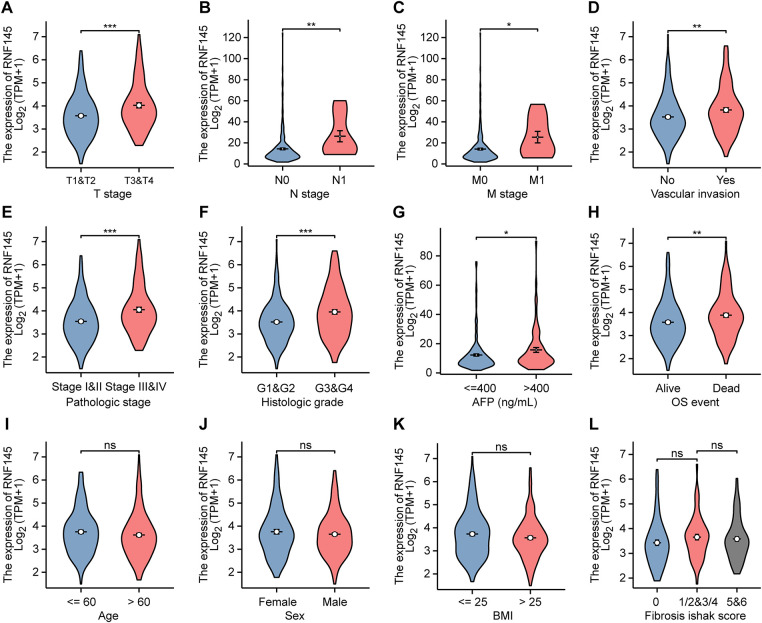
Expression of RNF145 was correlated with clinicopathological features. According to data from the TCGA database, the relation between mRNA expression of RNF145 and T stage (**A**), N stage (**B**), M stage (**C**), vascular invasion (**D**), pathological stage (**E**), histological grade (**F**), AFP (**G**), OS event (**H**), age (**I**), sex (**J**), BMI (**K**) and Fibrosis ishak score (**L**). *p < 0.05, **p < 0.01, ***p < 0.001, ns: no significant

We analyzed HCC samples for their RNF145 expression and acquired clinical data. T stage, N stage, M stage, vascular invasion, pathological stage, histological grade, AFP, and OS event were related to the expression level of RNF145 (Fig. 2A–H), while there was no convincing evidence that the expression level of RNF145 correlated with age, sex, BMI and fibrosis ishak score (Fig. 2I–L). A more detailed description of the above clinicopathological features of the patients with HCC was presented in a baseline data sheet (Table 1). In short, patients who were in a more advanced cancer stage or grade tended to express a higher level of RNF145.

**Table 1:** Baseline data table of HCC patients in TCGA

Characteristics	RNF145_Low	RNF145_High	*p* value
n	179	179	
Age, n (%)			0.112
<=60	78 (21.8%)	93 (26.0%)	
>60	101 (28.2%)	86 (24.0%)	
T Stage, n (%)			0.031
T1	104 (29.1%)	76 (21.2%)	
T2	37 (10.3%)	51 (14.2%)	
T3	32 (8.9%)	45 (12.6%)	
T4	6 (1.7%)	7 (2.0%)	
N Stage, n (%)			0.007
N0	121 (46.0%)	130 (49.4%)	
N1	1 (0.4%)	11 (4.2%)	
M Stage, n (%)			0.226
M0	128 (46.9%)	136 (49.8%)	
M1	2 (0.7%)	7 (2.6%)	
AFP (ng/mL), n (%)			0.048
<=400	118 (42.3%)	94 (33.7%)	
>400	28 (10.0%)	39 (14.0%)	
Grade, n (%)			0.070
G1	34 (9.6%)	20 (5.6%)	
G2	90 (25.3%)	82 (23.0%)	
G3	50 (14.0%)	68 (19.1%)	
G4	5 (1.4%)	7 (2.0%)	
Stage, n (%)			0.036
Stage I	97 (28.8%)	72 (21.4%)	
Stage II	35 (10.4%)	47 (13.9%)	
Stage III	33 (9.8%)	49 (14.5%)	
Stage IV	2 (0.6%)	2 (0.6%)	
OS Status, n (%)			0.186
0	121 (33.8%)	109 (30.4%)	
1	58 (16.2%)	70 (19.6%)	

Note: AFP, Alpha Fetoprotein; OS, Overall Survival; HCC, Hepatocellular Carcinoma; RNF145, Ring Finger Protein 145. *p* < 0.05 was considered statistically significant.

### 3.3 High Expression of RNF145 Is Associated with Poor Prognosis of HCC

**Figure 3:**
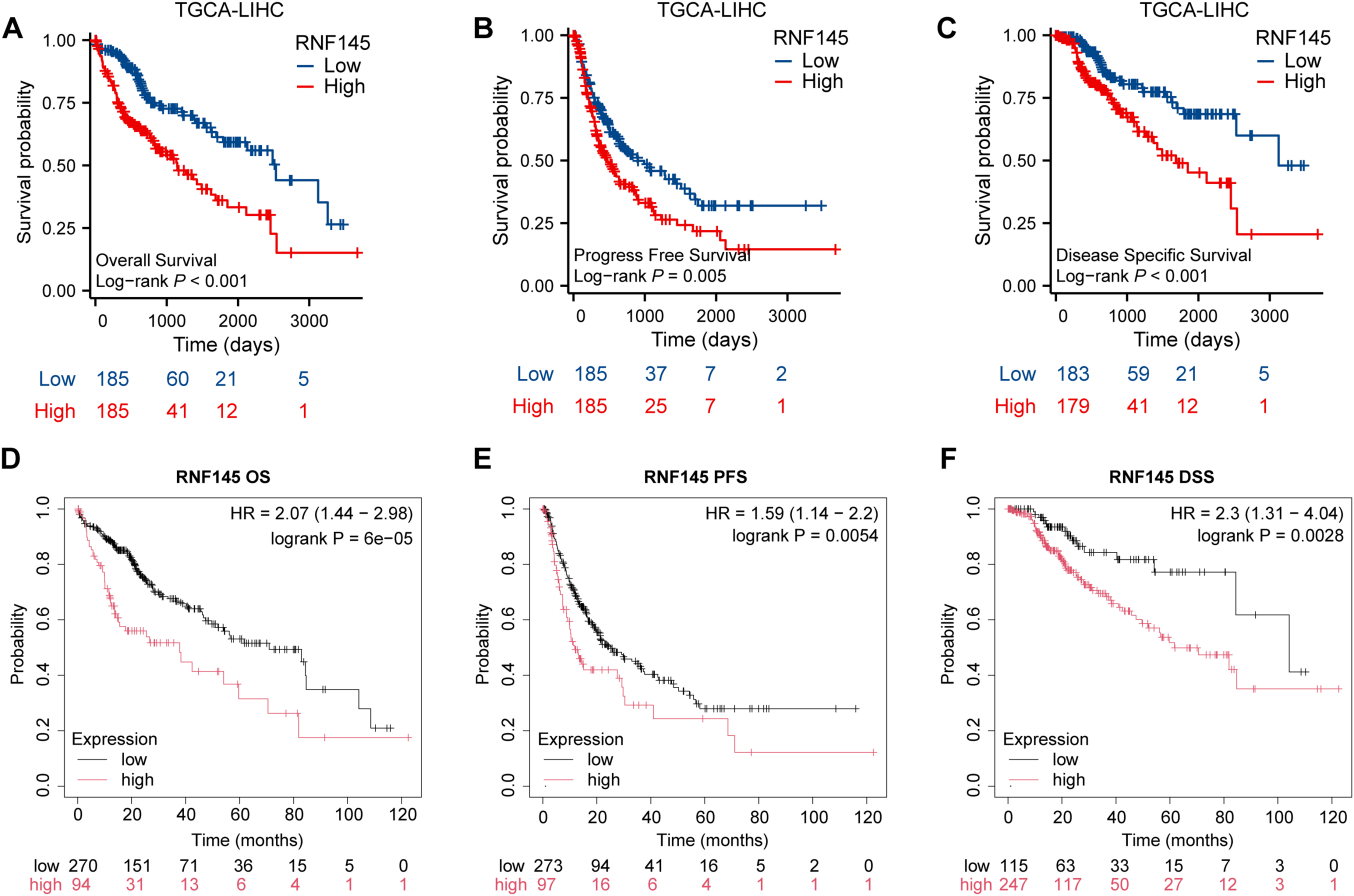

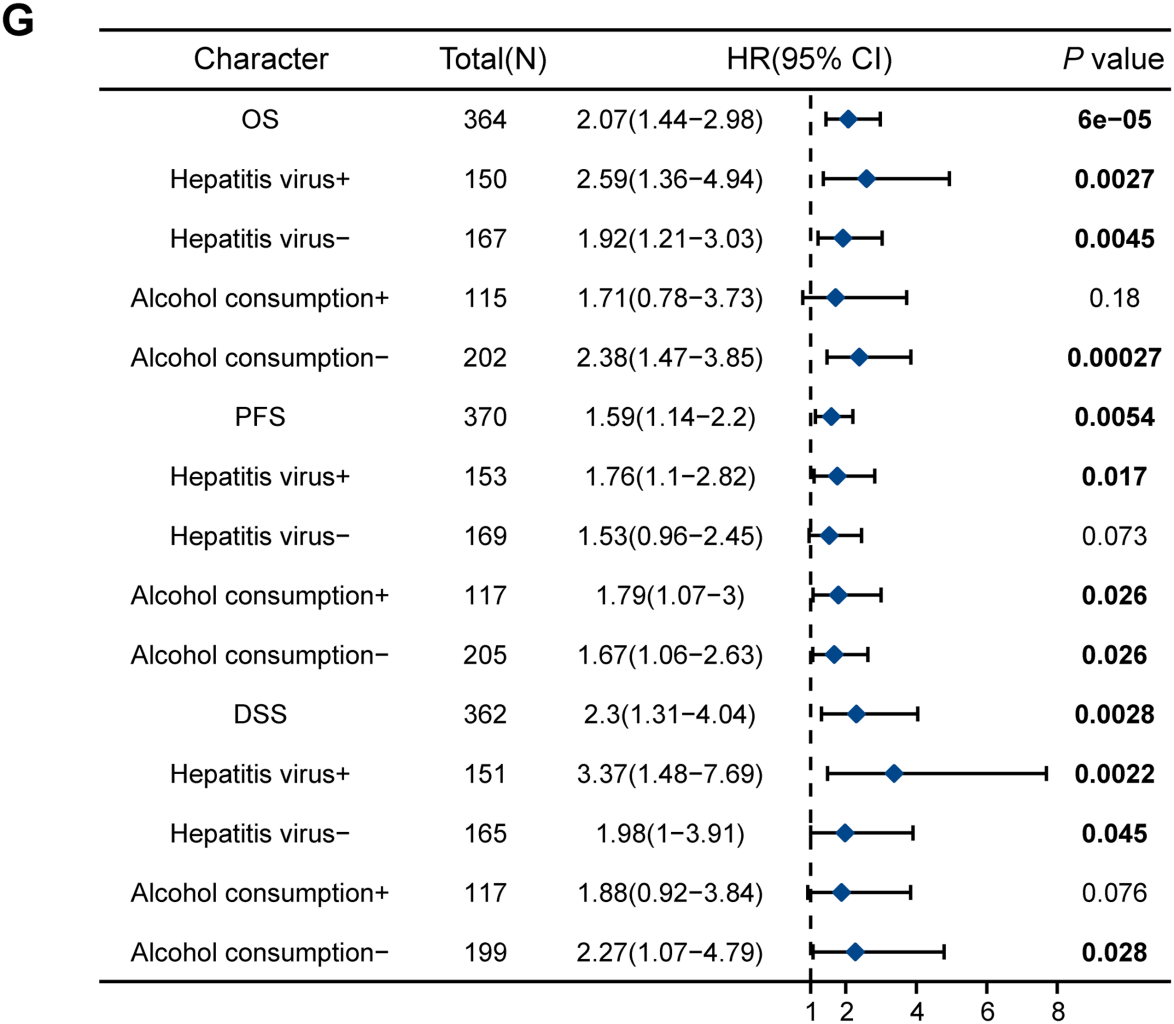
Kaplan–Meier survival curves were used to investigate the prognostic value of RNF145 expression in HCC. Kaplan–Meier survival curves of OS (**A**), PFS (**B**), and DSS (**C**) in the TGCA database. Kaplan–Meier survival curves of OS (**D**), PFS (**E**), and DSS (**F**) in the online database. (**G**) Forest plot summarizing subgroup analyses based on hepatitis virus infection and alcohol consumption status, showing hazard ratios for OS, DSS, and PFS. “+” indicated the infection of the hepatitis virus or alcohol consumption by the patient, while “−” indicated that the patient had not been infected with the hepatitis virus or did not consume alcohol

Kaplan–Meier survival curves were used to investigate the prognostic value of RNF145 expression in HCC. According to the results, patients with HCC with higher RNF145 expression showed a lower OS (*p* < 0.001), PFS (*p* = 0.005) and DSS (*p* < 0.001) (Fig. 3A–C). The online database Kaplan-Meier Plotter (https://kmplot.com/analysis/, accessed on 01 January 2025), which contains RNA-seq data from over 300 HCC patients, was used to further validate the above results [21]. The results also supported that patients with high expression of RNF145 in HCC have worse OS (*p* < 0.001), PFS (*p* = 0.0054) and DSS (*p* = 0.0028) (Fig. 3D–F). Hepatitis virus infection and alcohol consumption are common risk factors for liver cancers; Therefore, we further explored whether RNF145 expression was associated with OS, DSS, and PFS in a subgroup of patients with or without hepatitis virus infection and alcohol consumption using the data from the Kaplan-Meier Plotter database. Higher expression of RNF145, indicating poorer OS, DSS, and PFS was observed in patients with hepatitis virus infection. Among patients without hepatitis virus infection, the expression of RNF145 correlated significantly with OS and DSS. Among alcohol consuming patients, only PFS was related to RNF145 expression, while among alcohol free cases, OS and PFS were statistically significant. The above results were summarized in a forest diagram (Fig. 3G).

### 3.4 Univariate and Multivariate Cox Regression Analysis

**Figure 4:**
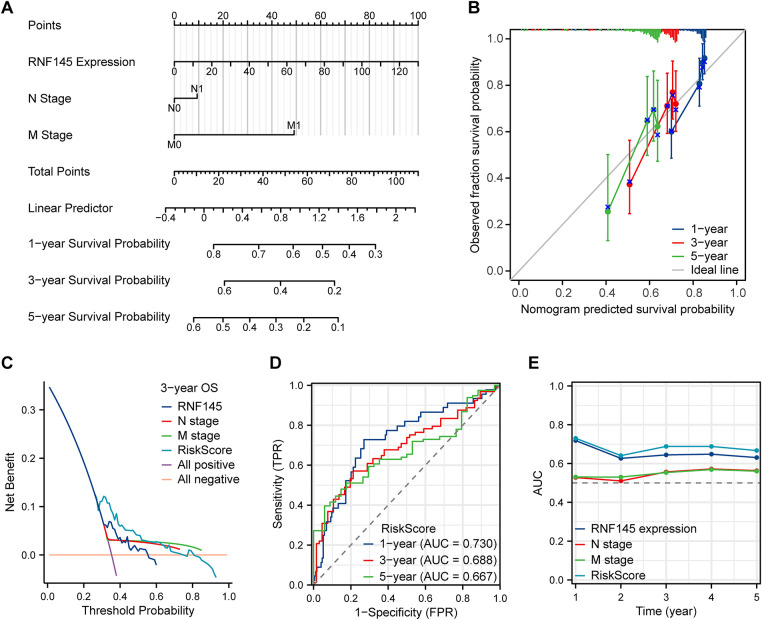
The nomogram and its performance in predicting 1-, 3-, and 5-year overall survival. A nomogram to predict the 1-, 3- and 5-year overall survival (**A**). Calibration curves for the nomogram (**B**). Decision curve analysis evaluating clinical utility (**C**). Time-dependent area under the curve analysis (**D**). Time-dependent area under the curve plot (**E**)

Univariate Cox regression analysis showed that the factors that might influence the survival of patients with HCC were T stage, N stage, M stage, pathological stage, and RNF145 expression (Table 2). Multivariate Cox regression analysis of the above factors indicated that the expression of RNF145 was an independent risk factor affecting the OS of patients with HCC (HR = 1.016, *p* = 0.008) (Table 2). Then, the three factors—RNF145 expression, N stage and M stage, that showed statistical significance in the multivariate Cox regression analysis were included and a nomogram was constructed (Fig. 4A). The prognostic calibration curve, decision curve analysis, time-dependent ROC curves and time-dependent AUC curves were used to evaluate the predictive efficacy of the nomogram for 1-, 3- and 5-year OS in HCC patients. The results show that this model has a higher predictive accuracy for the prognosis of HCC patients compared to RNF145 expression, N stage, and M stage (Fig. 4B–E).

**Table 2:** Table of univariate and multivariate Cox regression analysis

Characteristics	Total	Univariate analysis		Multivariate analysis	
		HR (95% CI)	*p* value	HR (95% CI)	*p* value
RNF145	358	1.019 (1.009–1.029)	<0.001	1.016 (1.004–1.029)	0.008
AFP (>400 vs. <=400)	278	1.202 (0.757–1.909)	0.435		
T Stage (T3&T4 vs. T1&T2)	358	2.573 (1.807–3.664)	<0.001	7.154 (0.772–66.257)	0.083
N Stage (N1 vs. N0)	263	3.245 (1.620–6.499)	<0.001	3.263 (1.215–8.762)	0.019
M Stage (M1 vs. N0)	273	4.979 (2.482–9.991)	<0.001	2.978 (1.196–7.417)	0.019
Grade (G3&G4 vs. G1&G2)	356	1.115 (0.778–1.599)	0.554		
Stage (III&IV vs. I&II)	337	2.488 (1.715–3.610)	<0.001	0.352 (0.037–3.323)	0.362
Age (>60 vs. <=60)	358	1.236 (0.870–1.756)	0.236		

Note: Abbreviatons, HR: Hazard Ratio; CI: Confidence Interval; AFP: Alpha Fetoprotein.

### 3.5 Enrichment Analysis of RNF145

**Figure 5:**
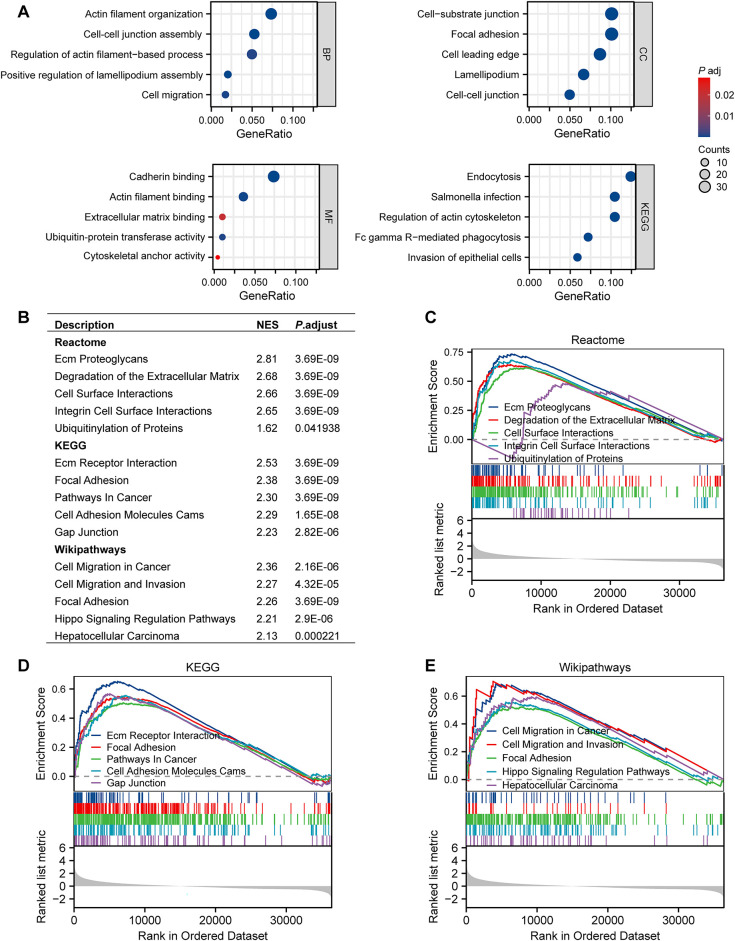
Functional enrichment analysis of RNF145 in HCC. BP, CC, MF, and KEGG pathways according to the expression of RNF145 in HCC (**A**). GSEA results from three commonly used gene sets (**B**). Reactome pathway enrichment (**C**). KEGG pathway enrichment (**D**). Wikipathways enrichment (**E**)

To explore the biological role of RNF145 in HCC, we performed GO and KEGG pathway analysis and GSEA of RNF145. The top four enrichment results of biological process (BP), cellular component (CC), molecular function (MF), and KEGG, respectively, were shown in bubble diagrams (Fig. 5A). The results of the three commonly used gene set analyses in GSEA are presented (Fig. 5B), including Reactome (Fig. 5C), KEGG (Fig. 5D) and Wikipathways (Fig. 5E), which are summarized and displayed separately. The enrichment analysis results indicated that RNF145 was significantly enriched in biological processes such as cell migration, invasion, cell adhesion and metastasis.

### 3.6 RNF145 Promotes Invasion and Migration of HCC Cells In Vitro

**Figure 6:**
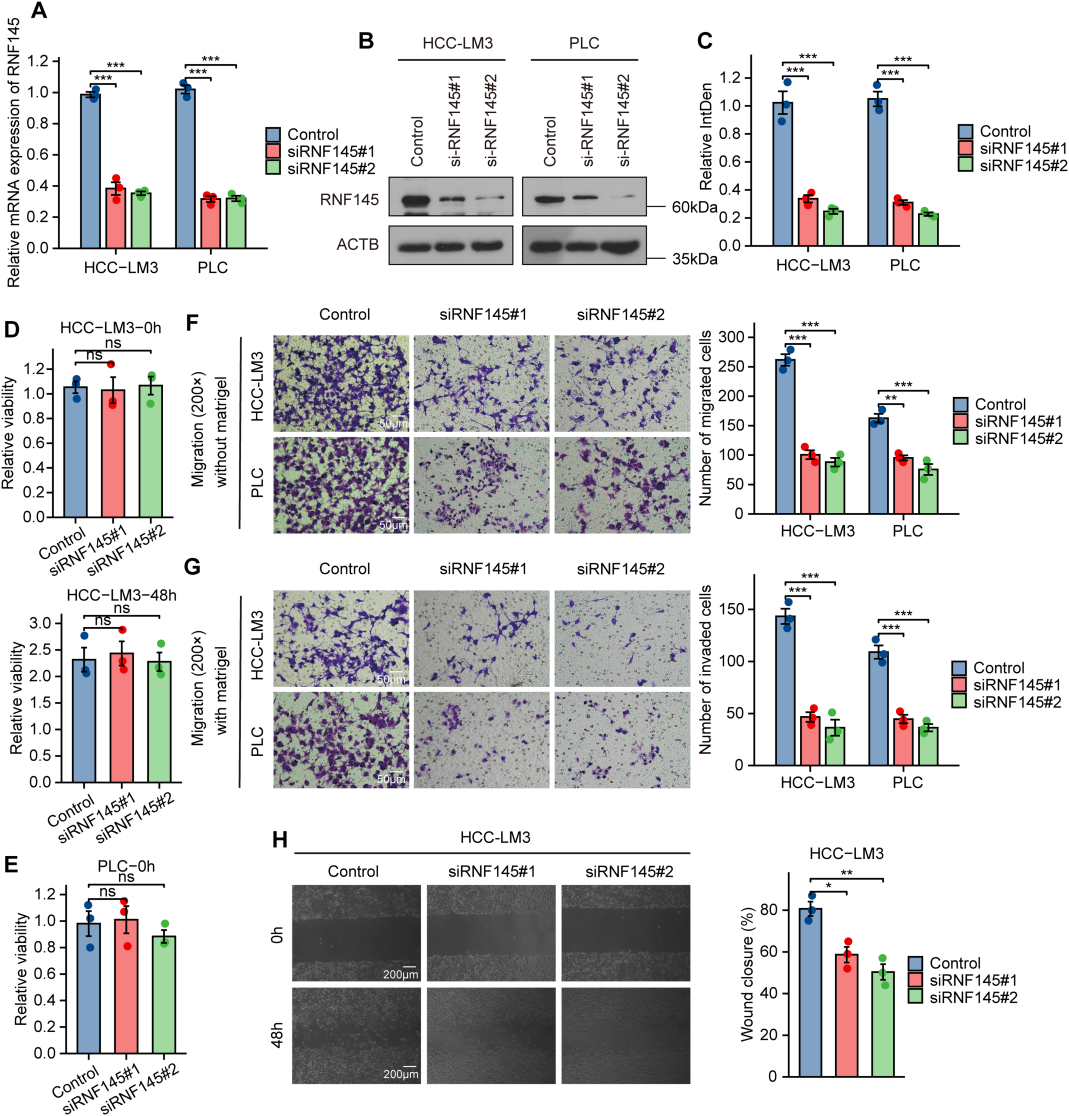

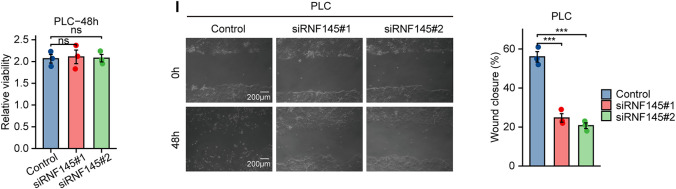
RNF145 promotes invasion and migration of HCC cells *in vitro*. Efficacy of two siRNAs was verified by qRT-PCR (**A**). Efficacy of two siRNAs was verified by western blot (**B**). Statistics of western blot using Image J (**C**). Cell viability of HCC-LM3 cells (**D**) and PLC (**E**) cells in 0 h and 48 h. Results of transwell migration (**F**) and invasion (**G**) assays in HCC-LM3 and PLC cells. Results of wound-healing assays in HCC-LM3 cells (**H**) and PLC cells (**I**). *p < 0.05, **p < 0.01, ***p < 0.001, ns: not significant

According to pathway enrichment analysis, RNF145 might be associated with pathways related to invasion and metastasis. To explore whether RNF145 was related to these biological behaviors, we first verified the efficacy of two siRNAs (Fig. 6A–C). The cell viability experiment showed that knocking down RNF145 did not affect the growth of the cells (Fig. 6D,E). Then we performed transwell migration and wound-healing assays. Transwell assays showed that the migratory and invasive ability of HCC-LM3 and PLC cells were significantly reduced when RNF145 was knocked down by the siRNAs (Fig. 6F,G). Analogously, the wounds healed much faster in the control group compared with those in the RNF145-si groups (Fig. 6H,I). Taken together, RNF145 promotes metastasis of HCC cells *in vitro*.

### 3.7 RNF145 Promotes Invasion and Migration by Facilitating PCDH9 Degradation

**Figure 7:**
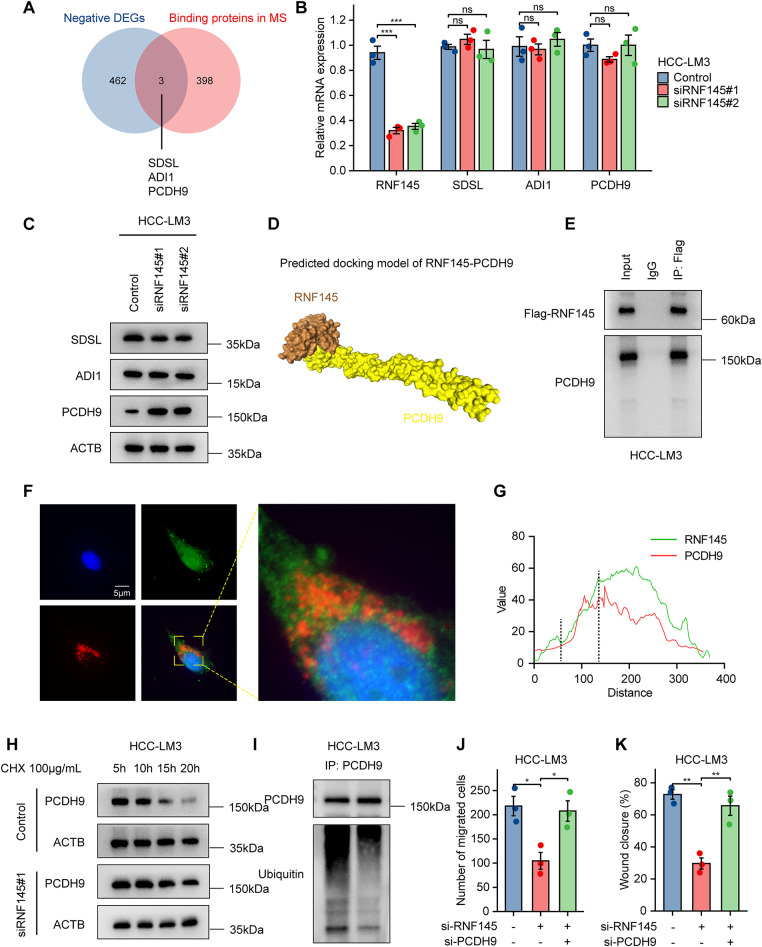
RNF145 promotes invasion and migration by facilitating PCDH9 degradation. Venn diagram of RNF145-interacting proteins from mass spectrometry and negatively correlated genes of RNF145 from TCGA (**A**). qPCR analysis of mRNA expression in control and RNF145-knockdown cells (**B**). Western blot of protein levels after RNF145 knockdown (**C**). HDOCK-predicted RNF145-PCDH9 binding mode (**D**). IP of Flag-RNF145 and HA-PCDH9 interaction (**E**). Representative immunofluorescence images of RNF145 and PCDH9 (**F**). Quantitative analysis of co-localization (**G**). CHX assay for PCDH9 half-life after RNF145 knockdown (**H**). Ubiquitination assay of PCDH9 under RNF145 knockdown (**I**). Rescue assays of migration (**J**) and invasion (**K**) in HCC-LM3 cells. *p < 0.05, **p < 0.01, ***p < 0.001, ns: not significant

To identify downstream molecules of RNF145, we employed a mass spectrometry database of RNF145 to identify the interacting proteins of RNF145 [27]. We found that SDSL, ADI1 and PCDH9 interacting with RNF145 were negatively correlated with RNF145 in the TGCA database using a Venn diagram (Fig. 7A). QRT-PCR analysis revealed that RNF145 knockdown did not significantly affect the mRNA levels of SDSL, ADI1, or PCDH9 (Fig. 7B). However, western blot demonstrated that the protein levels of SDSL and ADI1 remained unchanged, while PCDH9 was significantly upregulated (Fig. 7C). To predict the potential binding between RNF145 and PCDH9, we submitted the amino acid sequences of both proteins to the HDOCK online database (http://hdock.phys.hust.edu.cn/, accessed on 01 January 2025). The results suggested a potential binding mode between RNF145 and PCDH9, indicating a possible interaction between the two proteins (Fig. 7D). Subsequently, an exogenous co-immunoprecipitation assay was performed to investigate whether a direct binding relationship exists between RNF145 and PCDH9. The results demonstrated that Flag-tagged RNF145 binds with PCDH9 (Fig. 7E). Immunofluorescence analysis indicated that RNF145 and PCDH9 primarily co-localize in the cytoplasm (Fig. 7F,G). CHX assay revealed that RNF145 knockdown significantly extended the half-life of PCDH9 (Fig. 7H). After transfection with the Ubiquitin plasmid, co-immunoprecipitation assays were performed. Western blot analysis demonstrated that knockdown of RNF145 markedly decreased the ubiquitination of PCDH9 (Fig. 7I). Rescue experiments demonstrated that RNF145 knockdown restored the migratory and invasive ability of RNF145 knockdown in HCC-LM3 cells (Fig. 7J,K).

### 3.8 Genetic Alterations, DNA Methylation and Immune Infiltration Analysis of RNF145 in HCC

**Figure 8:**
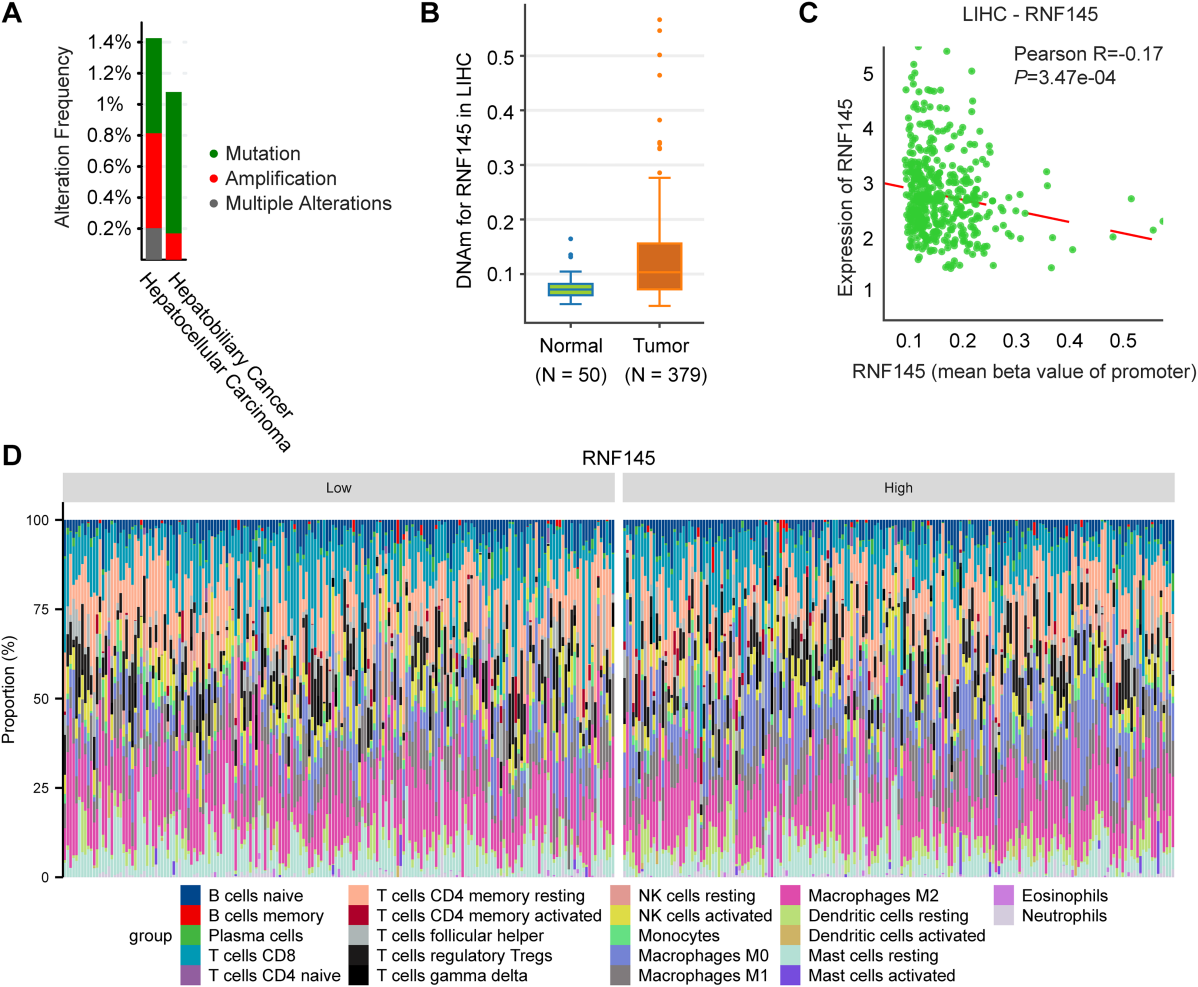

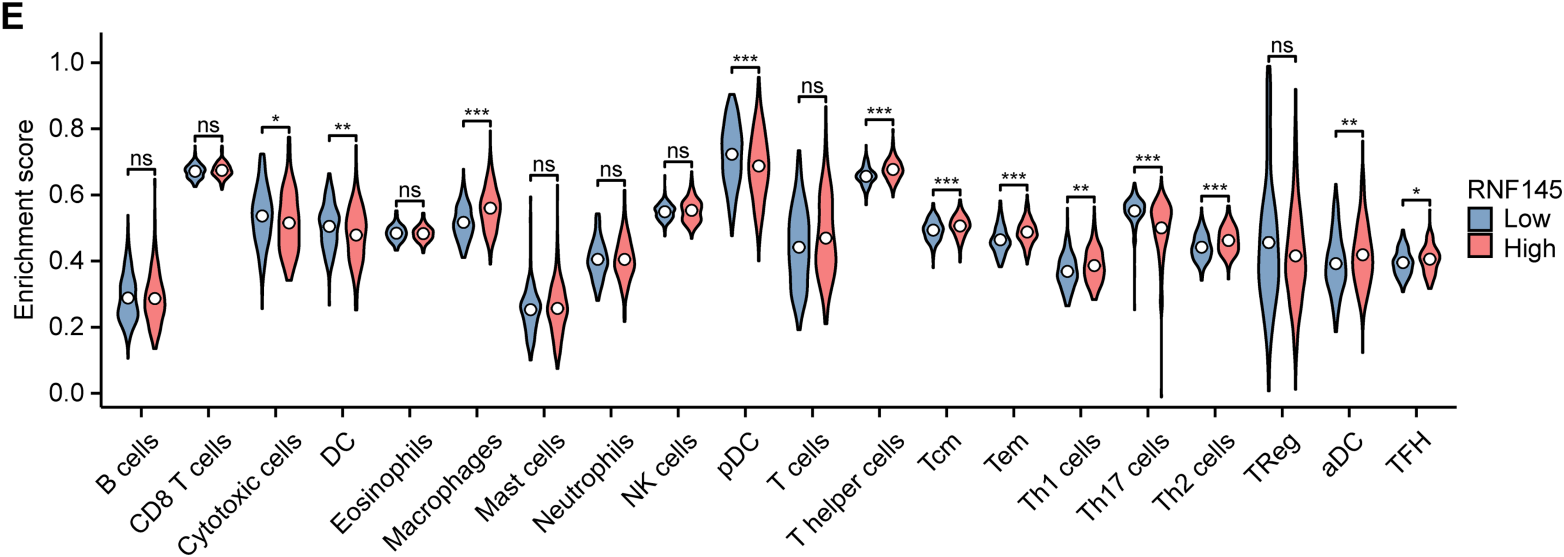
Genetic alterations, DNA methylation and immune infiltration analysis of RNF145 in HCC. Results of genetic alteration analysis using the data from the cBioPortal database (**A**). DNA methylation analysis of RNF145 in HCC (**B**). Correlation analysis of DNA methylation levels and RNF145 expression (**C**). Immune cell infiltration landscapes and statistics in the tissues of HCC patients with high and low expression of RNF145 (**D**,**E**). *p < 0.05, **p < 0.01, ***p < 0.001, ns: not significant

Finally, we investigated the potential reasons for the significant upregulation of RNF145 in HCC. The genetic alteration analysis using the data from the cBioPortal database indicates that the amplification rate of RNF145 in HCC is only 0.61% (Fig. 8A). DNA methylation analysis revealed a higher level of RNF145 methylation in HCC tissues compared to normal samples (Fig. 8B), with a significant negative correlation between RNF145 expression and its methylation status (Fig. 8C). These findings indicate that neither gene amplification nor methylation modifications account for RNF145 upregulation in HCC. Further analysis using the CIBERSORT algorithm delineated distinct immune cell infiltration patterns between RNF145 high-and low-expression groups in HCC. The RNF145 high-expression group exhibited significantly reduced infiltration of cytotoxic cells, dendritic cells, and Th17 cells, alongside increased infiltration of macrophages and Th2 cells (Fig. 8D,E). This pattern suggests that RNF145 overexpression may contribute to immunosuppression in the tumor microenvironment and reduced responsiveness to immunotherapy. This may provide a reference for the immune microenvironment state of HCC with high RNF145 expression and for the selection of patients for immunotherapy.

## 4 Discussion

HCC is one of the most common malignancies and places a huge health and financial burden on society worldwide. Despite continuous improvement in diagnostic methods and treatment, the prognosis of advanced HCC remains poor [28–31]. Finding effective prognostic markers and obtaining a better understanding of the potential mechanisms of progression of liver cancer are of great significance to improve the current treatment of patients with liver cancer.

Increasing evidence indicates that dysregulation of RING-finger ubiquitin-protein ligases (E3s) is involved in the development of HCC [32,33] and that RING-finger E3s might serve as oncoproteins or tumor suppressors in HCC under specific conditions [34,35]. RNF145 is a member of the RING-finger E3s [27]. However, it has been rarely reported in previous tumor-related studies. In this study, we explored the biological role of RNF145 in HCC through a bioinformatic analysis.

We verified that the mRNA and protein expression of RNF145 was higher in HCC tissues compared with that in normal liver tissues. High expression of RNF145 was related to a more advanced stage and a worse prognosis. Through univariate and multivariate Cox regression analysis, RNF145 expression was proven to be an independent risk factor affecting the overall survival of patients with HCC. The ROC curve indicated that RNF145 might be used as a valuable prognostic biomarker for HCC. GO and KEGG analyses showed that RNF145 might be involved with cell migration, invasion of epithelial cells and cadherin binding, almost all of which are closely related to invasion and metastasis [36,37]. To verify the results of enrichment analyses, we performed the transwell migration assays and wound-healing assays. RNF145 knockdown significantly reduced the invasion and migration of HCC cells.

The protocadherins (PCDHs), a key subfamily of calcium-dependent adhesion molecules, have been identified as potential tumor suppressor genes across multiple cancer types [9,38,39]. A previous study had shown that PCDH9 was downregulated in HCC and inhibits cell migration via GSK-3β activation [40]. Supporting this tumor-suppressive function, our study reveals that RNF145 facilitates the ubiquitination and subsequent degradation of PCDH9, which in turn promotes invasive and metastatic behavior in HCC cells. These results corroborate earlier evidence and nominate RNF145 as a putative therapeutic target and prognostic biomarker in HCC.

This study indicated that the amplification rate of RNF145 in liver cancer patients is very low. Therefore, further research is needed to clarify the reason for the abnormal upregulation of RNF145 in hepatocellular carcinoma. DNA methylation is a common epigenetic mechanism that plays a key role in regulating gene expression in all forms of cancer [41–43]. We demonstrated the relationship between the DNA methylation status of RNF145 and the prognosis of patients with HCC. The analysis results of this study suggested that genetic amplification or promoter DNA methylation may not be the primary mechanism underlying upregulation of RNF145 in HCC. The expression of genes in cells is subject to comprehensive regulation by multiple levels and various factors, including transcription, post-transcriptional, translation, and post-translational processes. A recent study reported that circZDBF2 upregulates RNF145 expression in oral squamous cell carcinoma through a competitive endogenous RNA mechanism [12]. Therefore, in order to clarify the reason for the dysregulation of RNF145 in HCC, further exploration and *in vitro* and vivo experiments are necessary. The correlation between RNF145 expression and the immune-related cells suggested that there might be potential regulatory mechanisms between them. The results might have reference value for future studies on immune-related markers of hepatocellular carcinoma.

RNF145 is a promising therapeutic target for this malignancy. Multiple protein-targeting strategies can be leveraged for its inhibition. Firstly, we can employ virtual screening of chemical libraries using the MTiOpenScreen platform and AutoDock Vina to identify potential compounds that target RNF145 [44]. High-throughput screening represents another viable approach to discover specific binders to RNF145, particularly within its RING domain, which is responsible for E2 ubiquitin-conjugating enzyme recruitment [45]. Concurrently, disrupting specific interaction of RNF145 and PCDH9 may enable the development of highly selective inhibitor. Moreover, given the demonstrated success of PROTAC technology for E3 ligases, developing RNF145-directed PROTACs offers a promising alternative strategy [46]. This bifunctional approach may overcome resistance mechanisms commonly associated with conventional inhibitors. Throughout the development process, rigorous assessment of compound selectivity, metabolic stability, and *in vivo* efficacy will be essential to translate these strategies into viable therapeutic candidates for hepatocellular carcinoma.

However, this study has several limitations. Due to certain experimental constraints, more compelling *in vivo* validation was not performed to substantiate the conclusions derived from *in vitro* cellular experiments. Furthermore, the specific binding sites between RNF145 and PCDH9 were not explored, and the upstream mechanisms underlying RNF145 overexpression in HCC remain unclear. It is therefore imperative that future research comprehensively elucidates these remaining questions.

## 5 Conclusion

In summary, this study demonstrates that RNF145 is upregulated in HCC and serves as a potential prognostic biomarker. Functional experiments confirmed that RNF145 promotes migration, invasion, and metastatic progression in HCC cells. Mechanistically, RNF145 facilitates the ubiquitination and degradation of PCDH9, revealing a novel RNF145-PCDH9 regulatory axis critical for HCC metastasis. These findings nominate RNF145 as a promising therapeutic target for future investigations.

## Data Availability

The datasets referred to in this study can be found in online repository/repositories. Further inquiries can be directed to the corresponding authors.
